# Obese Adipose Tissue Secretion Induces Inflammation in Preadipocytes: Role of Toll-Like Receptor-4

**DOI:** 10.3390/nu12092828

**Published:** 2020-09-16

**Authors:** Mariana Renovato-Martins, Catharina Moreira-Nunes, Georgia C. Atella, Christina Barja-Fidalgo, João Alfredo de Moraes

**Affiliations:** 1 Departamento de Biologia Celular e Molecular, Instituto de Biologia, Universidade Federal Fluminense, Niterói 24020-140, Brazil; 2 Programa de Pesquisa em Farmacologia e Inflamação, Instituto de Ciências Biomédicas, Universidade Federal do Rio de Janeiro, Rio de Janeiro 21941-902, Brazil; catharinanunes@hotmail.com (C.M.-N.); joaomoraes@icb.ufrj.br (J.A.d.M.); 3 Instituto de Bioquímica Médica Leopoldo De Meis, Universidade Federal do Rio de Janeiro, Rio de Janeiro 21941-902, Brazil; atella@bioqmed.ufrj.br; 4 Instituto Nacional de Ciência e Tecnologia em Entomologia Molecular- INCT-EM, Universidade Federal do Rio de Janeiro, Rio de Janeiro 21940-590, Brazil; 5 Departamento de Biologia Celular, IBRAG, Universidade do Estado do Rio de Janeiro, Rio de Janeiro 20551-030, Brazil; barja-fidalgo@uerj.br

**Keywords:** adipose tissue, preadipocyte, TLR4, inflammation, free fatty acid, obesity

## Abstract

In obesity, the dysfunctional adipose tissue (AT) releases increased levels of proinflammatory adipokines such as TNFα, IL-6, and IL-1β and free fatty acids (FFAs), characterizing a chronic, low-grade inflammation. Whilst FFAs and proinflammatory adipokines are known to elicit an inflammatory response within AT, their relative influence upon preadipocytes, the precursors of mature adipocytes, is yet to be determined. Our results demonstrated that the conditioned medium (CM) derived from obese AT was rich in FFAs, which guided us to evaluate the role of TLR4 in the induction of inflammation in preadipocytes. We observed that CM derived from obese AT increased reactive oxygen species (ROS) levels and NF-ĸB nuclear translocation together with IL-6, TNFα, and IL-1β in 3T3-L1 cells in a TLR4-dependent manner. Furthermore, TLR4 signaling was involved in the increased expression of C/EBPα together with the release of leptin, adiponectin, and proinflammatory mediators, in response to the CM derived from obese AT. Our results suggest that obese AT milieu secretes lipokines, which act in a combined paracrine/autocrine manner, inducing inflammation in preadipocytes via TLR4 and ROS, thus creating a paracrine loop that facilitates the differentiation of adipocytes with a proinflammatory profile.

## 1. Introduction

The obese state is described as the expansion of fat depots causing adipose tissue (AT) dysfunctionality, which is often characterized by low-grade inflammation in situ. This condition is highly correlated with the onset of obesity-related comorbidities including type 2 diabetes, insulin resistance, and several types of cancers [1]. It is consensual that AT-resident immune cells play a role in the regulation of this obesity-induced inflammation. Although different types of immune cells are found in AT, macrophages play a pivotal role in the establishment of inflammation, as they produce the most cytokines in response to obesity [2,3]. Despite the lack of information about the precise mechanism of the inflammatory response in expanding AT, it is suggested that adipocyte death, impairment of adipogenesis, fibrosis, hypoxia, oxidative stress, endoplasmic reticulum stress, and the dysregulation in free fatty acid (FFA) release contribute to this effect [4]. Once in a proinflammatory state, mature adipocytes secrete adipokines, such as TNFα, IL-6, and IL-1β [4] with visceral AT releasing high amounts of FFAs. Low-grade inflammation and the high rate of lipolysis are responsible for the negative metabolic consequences of fat accumulation in the body, such as insulin resistance, dyslipidemia, and lipotoxicity [5,6]. Even though the release of FFAs by AT ensures survival during prolonged food deprivation, it can be further augmented in obesity as a result of the spillover of lipoprotein lipase, an enzyme that processes triglyceride-rich proteins to release FFAs [7]. AT-derived cytokines, i.e., TNFα and IL-6, are known to stimulate lipolysis and promote the release of FFAs [6]. This can be further augmented when enlarged adipocytes release more FFAs together with a reduced FFA clearance [8].

The plasma FFA concentrations in obese individuals are commonly correlated with AT expansion [9,10], which can lead to the activation of TLR2 and TLR4, increasing NF-ĸB activity [11], eliciting the generation of proinflammatory cytokines [12]. TLRs are major upstream molecules in the activation of the IKKβ/NF-κB pathway, and their role in the development of obesity-induced inflammation has been studied. According to Shi et al., TLR4 is the molecular link between FFAs, inflammation, and the innate immune system [13]. In obese animal models, inhibition of the IKKβ/NF-κB pathway by pharmacological inhibitors of IKKβ, or by the genetic deletion of IKKβ, improves insulin resistance [14,15].

Recent studies have shown that the stromal vascular fraction (SVF) regulates the release of inflammatory mediators. Among the SVF, preadipocytes, the precursors of mature adipocytes, account for 15 to 50% of cells in human AT [5]. However, even though FFAs are known to elicit an inflammatory response in AT, the precise mechanism of how these FFAs act together in a paracrine manner on preadipocytes still needs to be determined.

Thus, we hypothesized that in response to obesity, there is an increased release of inflammatory mediators, as well as FFAs, which would act in a paracrine manner on preadipocytes to generate an inflammatory response, contributing to the establishment of a positive feedback loop in the low-grade inflammation of AT. We conducted this study to investigate whether the inflammatory mediators, as well and FFAs produced by the obese AT could act in a paracrine manner on preadipocytes to generate an inflammatory response, contributing to the establishment of a positive feedback loop in the low-grade inflammation of the AT. Understanding how the endocrine and immune functions interconnect in AT may pave the way for the development of new strategies for the treatment of obesity and its associated comorbidities.

## 2. Materials and Methods

### 2.1. Reagents

Benzamidine, bovine serum albumin (BSA), EDTA, HEPES, leupeptin, phenylmethylsulfonyl fluoride (PMSF), soybean trypsin inhibitor, and trypsin were obtained from Sigma-Aldrich (St. Louis, MO, USA). TLR4 antagonist TAK-242 (TAK) was obtained from the Cayman Chemical Company (Ann Arbor, MI, USA). Dulbecco’s modified Eagle’s medium (DMEM) and fetal bovine serum (FBS) were acquired from GIBCO (Carlsbad, CA, USA). Ready-To-Glow pNF-κB-secreted luciferase reporter system was obtained from Clontech (Mountain View, CA, USA). Antibodies anti-C/EBPα and anti-PPARγ were purchased from Santa Cruz Biotechnology (Dallas, TX, USA). Antibody anti-actin was purchased from Abcam (Cambridge, UK). Enzyme-linked immunosorbent assay (ELISA) kits (IL-6, IL-1β, Leptin, and TNFα) were purchased from Peprotech (Rocky Hill, NJ, USA). The adiponectin ELISA kit was purchased from R&D systems (Minneapolis, MN, USA). CM-H_2_DCFDA (DCF) was from Life Technologies (Carlsbad, CA, USA). The enhanced chemiluminescence (ECL) and BCA protein assay kit were purchased from Pierce Biotechnology (Waltham, MA, USA).

### 2.2. Obesity Animal Model

Animal experiments were performed in strict accordance with the recommendations of the 1964 Declaration of Helsinki. The animal study was approved by the Committee on the Ethics of Animal Experiments of the Federal University of Rio de Janeiro (UFRJ) (CEUA 042/2016). We obtained C57BL/6J mice from the animal facilities of CEMIB/UNICAMP (São Paulo, Brazil). Animals were housed (four mice per cage) in a temperature-controlled room (25 ± 1 °C) and 12 h artificial light-dark-cycle. Upon weaning, male mice were randomly divided into two groups and fed according to AIN-93M recommendations: Standard chow (control group: 3.9 kcal/g of chow; 13% of energy derived from fat) or high-fat diet chow (high-fat diet group (HFD): 4.7 kcal/g, calorically enhanced by lard; 45% of energy derived from fat) until 90 days of age, when they were euthanized. Further information about the diet composition is shown in Table S1. The body weight of each animal was evaluated at 90 days of age.

### 2.3. AT Explant Culture

Epididymal AT obtained from control and obese mice was rinsed and cleaned with a phosphate-buffered saline. The explant culture was performed as previously described [16]. Briefly, 100 mg of AT explants obtained from mouse AT was incubated in 1 mL of DMEM supplemented with 1% FBS at 37 °C for 24 h. After this time, supernatants (conditioned medium [CM]) were aspirated on ice and centrifuged at 350× *g* for 10 min at 4 °C. The supernatants were collected (CM) and stored at −80 °C for further experiments.

### 2.4. Gas Chromatography-Mass Spectrometry (GC–MS)

The content of long-chain fatty acids in the CM was analyzed in a volume corresponding to 500 μg of proteins using GC–MS, as previously described [17]. Lipid samples were dissolved in 1 mL toluene, and to this was added 2 mL of 1% sulfuric acid in methanol. After 24 h in a stoppered tube at 50 °C, 1 mL of 5% NaCl was added, and the required esters were extracted (2X) with 2 mL hexane and then removed in a stream of nitrogen. Dried fatty acid methyl esters (FAME) were suspended in 100 μL heptane. GC/MS analyses were carried out on a Shimadzu GCMS-QP2010 Plus system, using an HP Ultra 2 (5% phenyl-methylpolysiloxane) and Agilent (25 m × 0.20 mm × 0.33 μm). The splitless injector was set at 250 °C. Column temperature was programmed to increase from 40–160 °C at 30 °C/min, 160–233 °C at 1 °C/min, 233–300 °C at 3 °C/min, and held at 300 °C for 10 min. We used helium as the carrier gas with a linear velocity of 36.0 cm/s. Then, a volume of 2 mL of the sample was injected into the chromatograph. Electron ionization (EI-70 eV) and a quadrupole mass analyzer performed the analysis in scans from 40 to 440 amu. The interface was set at 240 °C and the ion source at 240 °C. The components were identified by comparing their mass spectra with those of the library NIST05 contained in the computer of the mass spectrometer. To confirm the identity of the peaks in the chromatogram by Supelco 37 Component FAME Mix (Sigma-Aldrich), we used retention indices. FFAs were quantified by determining peak-area ratios with the internal standards 9:0 and 19:0.

### 2.5. Cell Culture

3T3-L1 preadipocytes were obtained from the American Type Culture Collection (Rockville, MD, USA). The cells were cultured in DMEM containing 10% FBS, 100 µg/mL streptomycin, and 50 U/mL penicillin. The cells were incubated at 37 °C in a 5% CO_2_ atmosphere. The cells were passaged following dissociation with 0.1%/0.01% trypsin/EDTA, and after this process, the cells were seeded into new culture flasks for a maximum of five passages.

### 2.6. Reactive Oxygen Species (ROS) Production

3T3-L1 cells (5 × 10^3^ cells/well) were seeded in 96-well black plates overnight in DMEM containing 10% FBS. The cells were washed three times with PBS, then the medium was removed, and cells were incubated with DMEM containing 1% FBS and incubated for 1 h. To detect intracellular ROS, 3T3-L1 cells were loaded with DCF (10 µM) for 1 h and then washed to remove the excess probe. Cells were pretreated or not with TAK (1 µM) for 15 min and then incubated with Lean CM, Obese CM, or LPS 1 µg/mL for 2 h at 37 °C in a 5% CO_2_ atmosphere. CM-H_2_DCFDA fluorescence was monitored at an excitation of 495 nm and emission of 530 nm wavelengths. Fluorescence was quantified using the Flexstation™ multilabel plate reader (Molecular Devices, San Jose, CA, USA).

### 2.7. NF-ĸB Activation

3T3-L1 cells (2 × 10^5^ cells/well) were seeded in 24-well plates in DMEM containing 10% FBS overnight. The cells were washed three times with PBS, then the medium was removed, and cells were incubated with DMEM containing 1% FBS and incubated for 1 h. The cells were then washed three times with PBS and transfected with the NF-ĸB-responsive luciferase reporter construct (NF-ĸB pMetLuc 2) and its control plasmid (pMetLuc 2) in DMEM containing 1% FBS and incubated for 24 h. Cells were pretreated or not with TAK (1 µM) for 15 min and then incubated with Lean CM, Obese CM, or LPS 1 µg/mL for 2 h at 37 °C in a 5% CO_2_ atmosphere. The medium containing luciferase was collected for each treatment and was incubated with luciferin. Luminescence emitted from the luciferin cleavage was monitored using the Flexstation™ multilabel plate reader (Molecular Devices, San Jose, CA, USA).

### 2.8. Immunofluorescence Microscopy

3T3-L1 cells were plated (5 × 10^4^ cells) on glass coverslips in DMEM containing 10% FBS and grown on glass coverslips at 37 °C in a 5% CO_2_ atmosphere. The next day, the cells were washed with PBS, then cells were incubated with a serum-free medium, and after 30 min, the cells were stimulated with Lean CM, Obese CM, or LPS 1 µg/mL for 2 h. The monolayers were washed with PBS, and the cells were fixed with 4% paraformaldehyde/4% sucrose in PBS. After 20 min, the cells were blocked with 5% BSA in PBS for 30 min. Then, cells were washed three times with PBS and incubated with phalloidin-TRITC (1:1000) at room temperature. After 2 h, 3T3-L1 cells were washed three times with PBS and incubated for 24 h with anti-NF-κB (1:200) at 4 °C. The cells were rinsed with 0.1% Tween in TBS and incubated for 1 h at room temperature with a FITC-conjugated secondary antibody (1:200). Coverslips were mounted on a slide with the use of DAPI Prolong for nuclear staining before examination under an epifluorescence microscope (BX40 Olympus). The images were analyzed using ImageJ (NIH).

### 2.9. ELISA

3T3-L1 cells (2 × 10^5^ cells/well) were seeded in 24-well plates in DMEM containing 10% FBS overnight. The cells were washed three times with PBS, and the medium was replaced with DMEM containing 1% FBS and incubated for 1 h. Cells were pretreated or not with TAK (1 µM) for 15 min and then incubated with Lean CM, Obese CM, or LPS 1 µg/mL at 37 °C in a 5% CO_2_ atmosphere for 3 h. The medium was then replaced with DMEM containing 1% FBS and incubated for 24 h to quantify IL-6, IL-1β, and TNF-α levels. To quantify adiponectin, IL-6, IL-1β, leptin, and TNF-α levels after differentiation, cells were pretreated or not with TAK (1 µM) for 15 min and then incubated with Lean CM, Obese CM, or Differentiation Medium (dexamethasone 1 µM, isobutylmethylxanthine 0.5 mM, and insulin 5 µg/mL) at 37 °C in a 5% CO_2_ atmosphere for six days. The medium was then replaced with DMEM containing 1% FBS and incubated for 24 h. These adipokines were quantified in supernatants using a sandwich ELISA kit. All procedures were performed according to the manufacturer’s instructions.

### 2.10. Cellular Extract

3T3-L1 cells (1 × 10^6^ cells/well) were cultured on 6-well plates with DMEM 10% FBS for 24 h. Then, the cells were washed with PBS and incubated with serum-free DMEM for 1 h. Groups were pretreated or not with TAK (1 µM) for 15 min and then incubated with Lean CM, Obese CM, or Differentiation Medium (dexamethasone 1 µM, isobutylmethylxanthine 0.5 mM, and insulin 5 µg/mL) for 48 h at 37 °C in a 5% CO_2_ atmosphere. Then, 3T3-L1 cells were lysed in a lysis buffer (benzamidine 1 mM, EDTA 10 mM, HEPES 50 mM, pH 6.4, MgCl_2_ 1 mM, 1 % Triton X-100, DNase 1 µg/mL, Rnase 0.5 µg/mL, PMSF 1 mM, leupeptin 1 µg/mL, and soybean trypsin inhibitor 1 µg/mL).

### 2.11. Western Blot Analysis

The total protein in the cell extracts was determined by the BCA method. Cell lysates were denatured in a sample buffer (Tris·HCl 50 mM, 10% glycerol, pH 6.8, 1% SDS, 5% 2-ME, and 0.001% bromophenol blue) and heated in a boiling water bath for 3 min. We loaded 30 µg of proteins from each sample onto electrophoresis gels. Next, the proteins were electroblotted from the gels to nitrocellulose membranes and blocked with 5% BSA containing 0.1% Tween in TBS. The blocked membranes were incubated overnight at 4 °C with primary antibodies. After washing in Tween-TBS, the membranes were incubated for 2 h with a peroxidase-conjugated secondary antibody. The bands were visualized using ECL and quantified by densitometry using the ImageJ software (NIH). The results are expressed as fold increase compared to the control after normalization with the housekeeping protein actin.

### 2.12. Adipogenesis Quantification

3T3-L1 cells (1 × 10^3^ cells/well) were seeded in 96-well plates in DMEM containing 10% FBS overnight. The cells were washed three times with PBS, and the medium was replaced with DMEM containing 1% FBS and incubated for 1 h. Cells were pretreated or not with TAK (1 µM) for 15 min and then incubated with Lean CM, Obese CM, or Differentiation Medium (dexamethasone 1 µM, isobutylmethylxanthine 0.5 mM, and insulin 5 µg/mL) at 37 °C in a 5% CO_2_ atmosphere. The medium was replaced every three days. After seven days, the medium was removed and cells were fixed with formalin 10%. After 30 min, the cells were washed and incubated with oil red O for 20 min. Then, the cells were washed and treated with 70% isopropanol, and the absorbance was quantified at 492 nm using the Flexstation™ multilabel plate reader (Molecular Devices, San Jose, CA, USA).

### 2.13. Statistical Analysis

The data are expressed as means ± standard error. Statistical significance was assessed by ANOVA, followed by the Bonferroni t-test. For all analyses, a *p*-value < 0.05 was considered statistically significant.

## 3. Results

### 3.1. AT from Obese Animals Released Increased Amounts of FFAs

**Figure 1:**
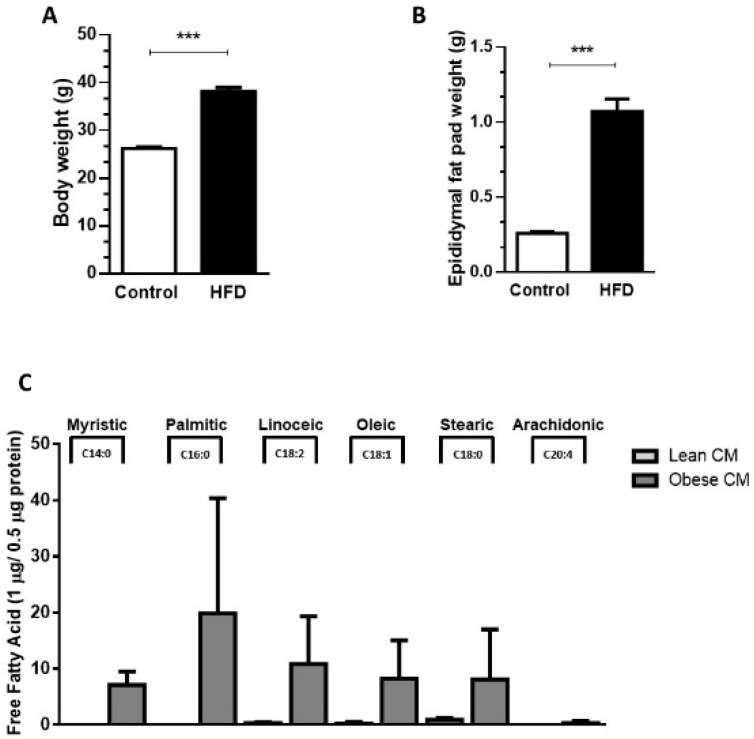
Male mice were randomly housed in cages (*n* = 4 animal per cage) and, after weaning were fed either with regular control chow (CTL; 13% of energy derived from fat) or high-fat-diet (HFD; 45% of energy derived from fat) until 90 days of age. (**A**) Body weight and epididymal fat pad (**B**) were measured after 90 days. Results are representative of 28 at least 30 mice. Data are expressed as means ± SEM. *** *p* < 0.005 vs. control. (**C**). Epididymal adipose tissue depots were collected and maintained in culture in Dulbecco’s modified Eagle’s medium (DMEM) 1% FBS for 24 h. Then, the supernatant was collected, and free fatty acids were analyzed in gas chromatography-mass spectrometry (GC-MS). Results are representative of three different experiments.

Our results demonstrated that, compared to controls (lean), the 90-day-old obese mice showed a 45.7% increase in body weight (Figure 1A) and a 75% increase in epididymal fat pad weight (Figure 1B). The expansion in adiposity was accompanied by dramatic changes in the quality of FFAs released by the AT of obese mice. The analysis of FFAs present in the CM harvested from cultures of visceral AT showed that the AT from obese mice, but not lean mice, released myristic (14:0), palmitic (16:0), and arachidonic (20:4) acids. Furthermore, the AT from obese mice secreted increased quantities of linoleic (18:2; 36-fold), oleic (18:1; 33-fold), and stearic (18:2; 9-fold) acids in the CM, when compared to the AT from control mice (Figure 1C).

### 3.2. FFAs Released by Obese AT Induce ROS Production and NF-ĸB Translocation in Preadipocytes in a TLR4-Dependent Manner

**Figure 2:**
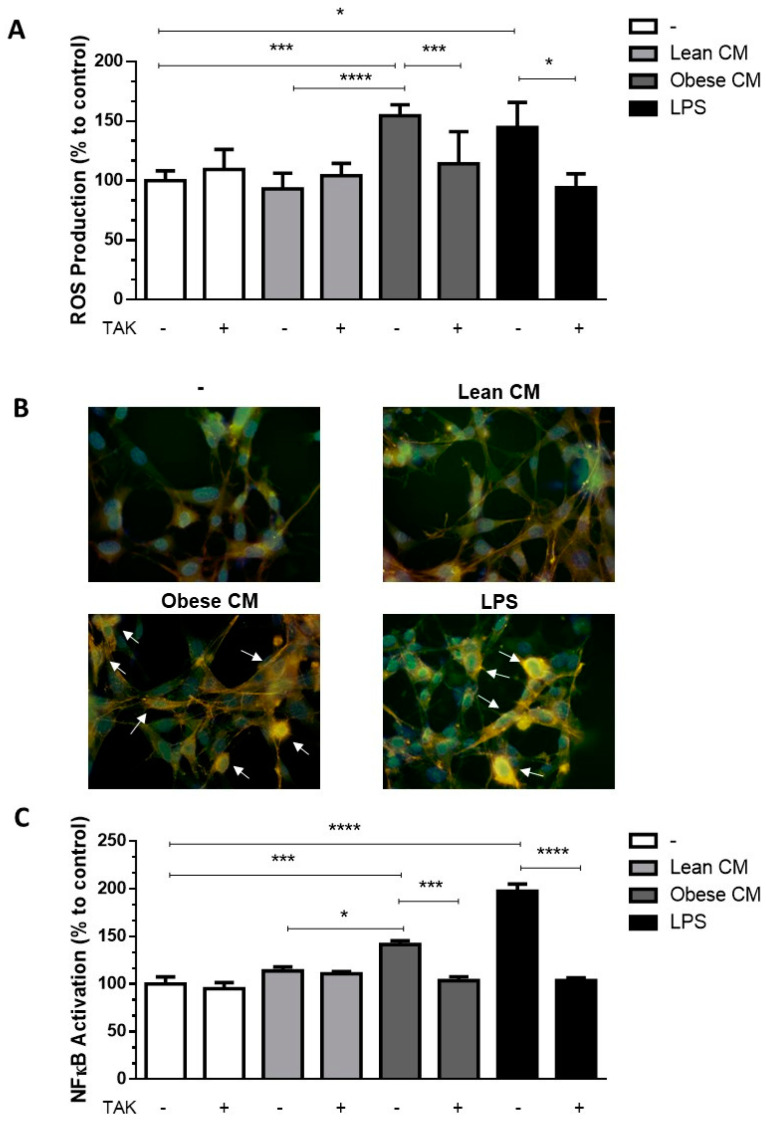
3T3-L1 cells were left untreated or were pre-incubated with TAK-242 (TAK) 1 µM for 15 min, at 37 °C/5% CO_2_. After pre-treatment, 3T3-L1 cells were treated or not with Lean Conditioned Medium (CM), Obese Conditioned Medium, or LPS 1 µg/mL for 2 h. (**A**). Reactive oxygen species (ROS) production was assessed using CM-H_2_DCFDA probe. (**B**). NF-κB translocation to nucleus was evaluated by immunofluorescence staining with anti-NF-κB-FITC (green), actin was visualized using phalloidin-rodhamin (red) and nuclei was visualized using DAPI (blue). Arrows indicate the NF-κB presence in nucleus. Results are representative of three different experiments. (**C**). NF-κB activation was evaluated by luciferase activity. (**A**,**C**). Results are representative of three independent experiments. Data are expressed as means ± SEM. * Represents *p* < 0.05, *** represents *p* < 0.005, **** represents *p* < 0.001. LPS—Lipopolysaccharide.

Once the CM from cultures of AT from obese animals was enriched with FFAs, we determined the cytotoxic potential of the CM from lean and obese AT, analyzing the viability of 3T3-L1 cells after 24 h of incubation with different concentrations of CM (10, 20, and 30% *v*/*v*) (Supplementary Materials Figure S1). Based on these results, the concentration of CM at 20% was utilized in all assays throughout the study. FFAs play a role in the recruitment of macrophages into AT [18]. Although the paracrine/autocrine effects of FFAs on adipocytes have been demonstrated [19], no experimental data on the effects of AT secretion on preadipocytes are available yet. We analyzed the ROS production in 3T3-L1 cells stimulated with CM derived from AT. It was observed that the CM derived from obese AT induced a 1.65-fold-increase in ROS production by 3T3-L1 cells when compared to that of control AT. As expected, LPS, used as a positive control, induced a greater increase in ROS levels in the preadipocytes. Additionally, we observed that when TLR4 was selectively inhibited by TAK, ROS production induced by obese AT CM or by LPS, significantly decreased to the control levels (Figure 2A).In order to prove that the CM was not free from LPS, we performed one experiment in the presence of polymyxin, which blocks the effects of LPS by binding to lipid A [20,21], and the results were similar to the CM itself (Figure S2A). We observed an increase in the expression of TLR4 mRNA in 3T3-L1 cells stimulated by CM derived from obese AT (Figure S3), suggesting that TLR4-dependent signaling may be increased in the preadipocytes.

In agreement with the data, we demonstrated that the CM derived from obese AT induced the nuclear translocation of NF-ĸB (Figure 2B) in 3T3-L1 cells, indicative of the activation of this pathway. This effect was eradicated by TLR4 signaling inhibition (Figure 2C).

### 3.3. Stimulation of Preadipocytes with CM Derived from Obese AT Increased Inflammatory Cytokines Released via TLR4 Signaling

**Figure 3:**
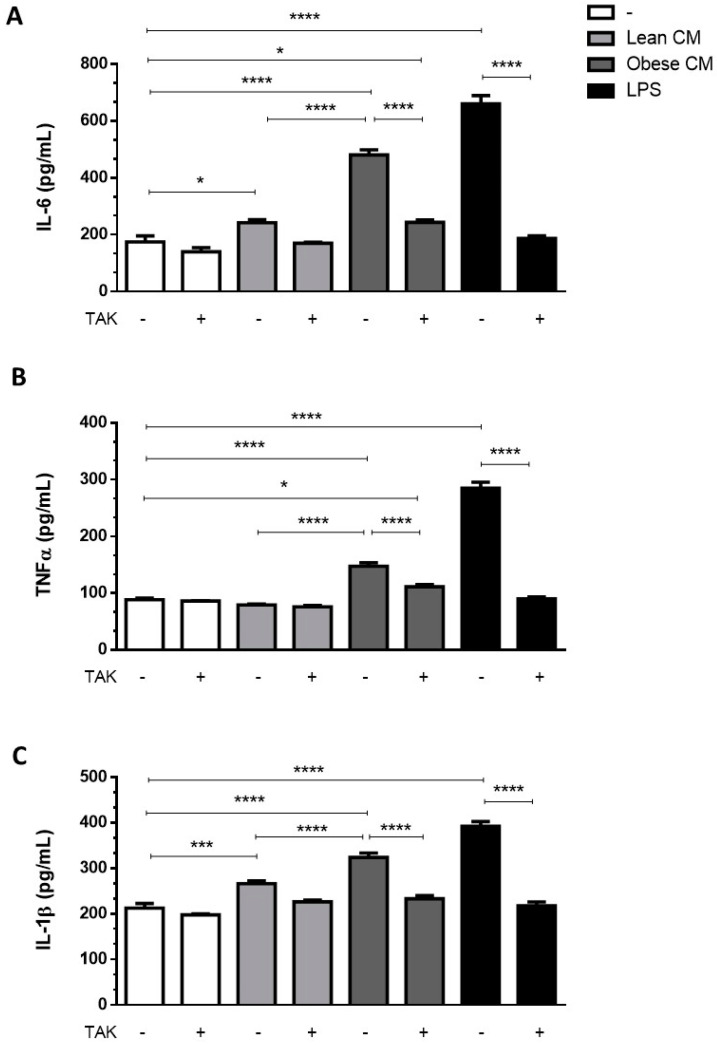
3T3-L1 cells were left untreated or were pre-incubated with TAK-242 (TAK) 1 µM for 15 min, at 37 °C/5% CO_2_. After pre-treatment, 3T3-L1 cells were treated or not with Lean Conditioned Medium (CM), Obese Conditioned Medium or LPS 1 µg/mL for 3 h. Then, the medium was removed, and cells were incubated with DMEM 1% FBS, for 21 h (to complete 24 h). IL-6 (**A**), TNFα (**B**), and IL-1β (**C**) were measured in supernatants using a sandwich enzyme-linked immunosorbent assay kit. Results are representative of three independent experiments. Data are expressed as means ± SEM. * Represents *p* < 0.05, *** represents *p* < 0.005, **** represents *p* < 0.001.

Adipocytes within AT are known to contribute to the low-grade inflammation apparent in obesity, by secreting increased amounts of proinflammatory cytokines [22]. To investigate whether preadipocytes may also contribute to this inflammatory profile in the obese AT microenvironment, we incubated 3T3-L1 cells with the CM derived from AT for 3 h and then analyzed the cytokines released by these cells in a CM-free medium. The results depicted in Figure 3 show that the cells primed with obese AT released increased amounts of IL6 (Figure 3A), TNF-α (Figure 3B), and IL-1β (Figure 3C) for the subsequent 24 h. The release of cytokines was prevented in the presence of TAK, a TLR4 signaling inhibitor (Figure 3A–C). Altogether, these results indicate that the contact of preadipocytes with the secretion released from obese AT induced, in a paracrine manner, a proinflammatory phenotype in preadipocytes within the AT, which is mediated by TLR4 signaling.

### 3.4. CM Derived from Obese AT Increased C/EBPα Expression in Preadipocytes via TLR4 Signaling

**Figure 4:**
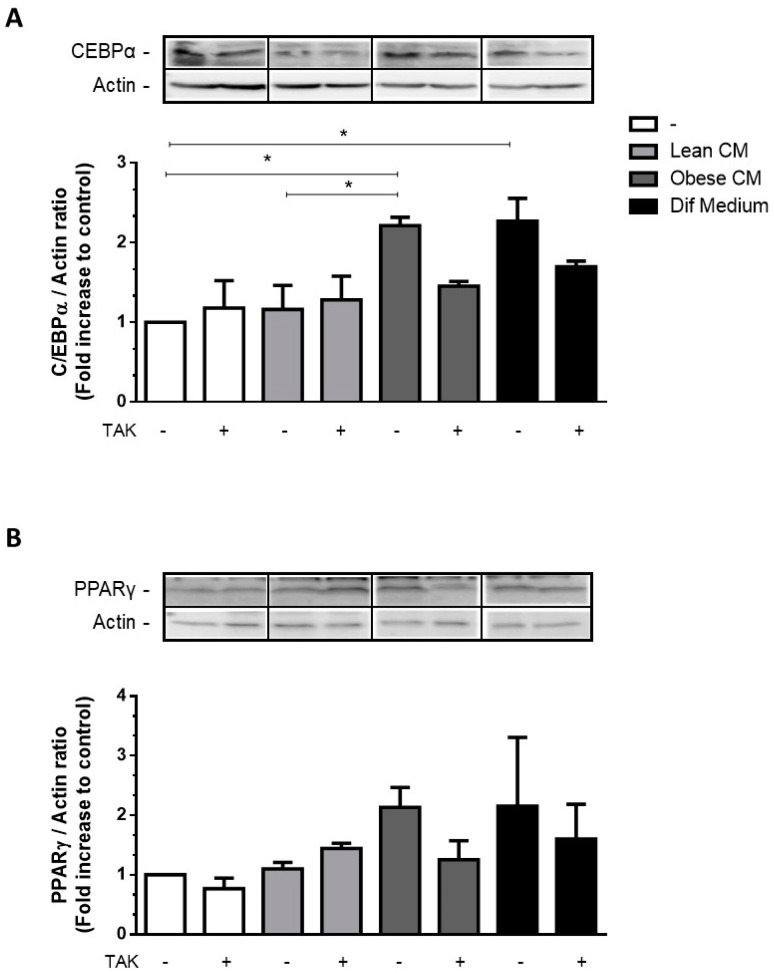
3T3-L1 cells were left untreated or were pre-incubated with TAK-242 (TAK) 1 µM for 15 min, at 37 °C/5% CO_2_. After pre-treatment, 3T3-L1 cells were treated or not with Lean Conditioned Medium (CM), Obese Conditioned Medium, or differentiation mix for 48 h. Thirty µg of proteins from cell lysates were subjected to Western blotting for C/EBPα (**A**) and PPARγ (**B**). Results are representative of three independent experiments. Data are expressed as means ± SEM. * Represents *p* < 0.05.

The transcription factors C/EBPα and PPARγ are key activators of adipogenesis reported to cooperate in the activation of a few adipocyte genes directly associated with the maturation of the adipocyte phenotype [23]. Most of the FFAs are among the compounds that are able to activate all three members of the PPAR family [24]. We have investigated the effect of CM derived from obese AT, rich in saturated and unsaturated FFAs, on the expression of adipogenic genes in 3T3-L1 cells. We showed that the stimulation of 3T3-L1 cells with the CM derived from obese AT increased the expression of C/EBPα (Figure 4A) and induced a trend of increase in PPARγ (Figure 4B) at levels comparable to those induced by the differentiation cocktail.

### 3.5. Preadipocytes Stimulated with the CM Derived from Obese AT Underwent Morphologic Changes and Lipid Accumulation in a TLR4-Dependent Manner

**Figure 5:**
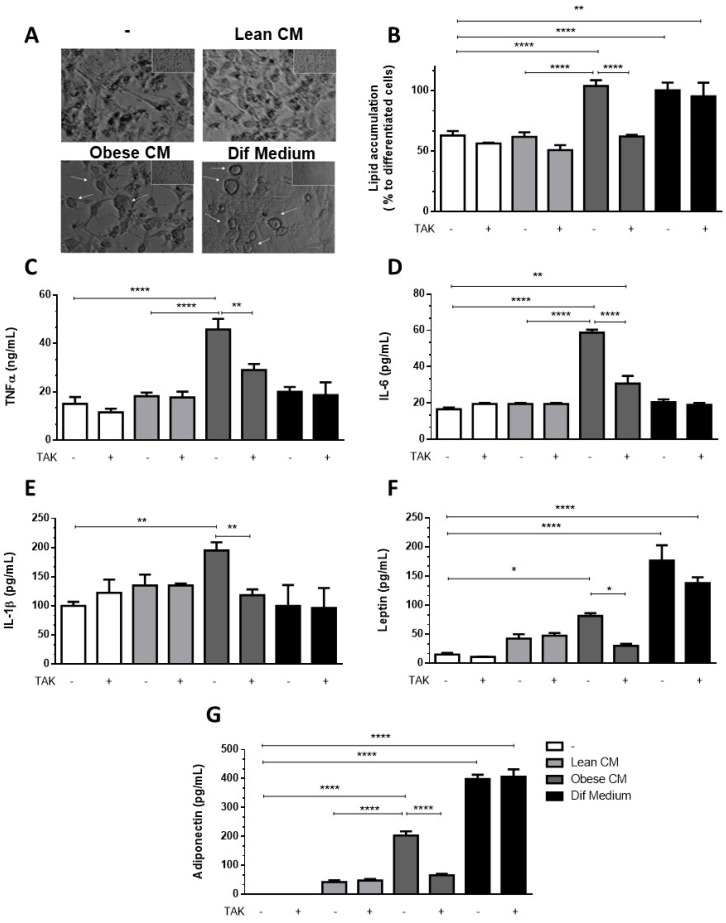
3T3-L1 cells were left untreated or were pre-incubated with TAK-242 (TAK) 1 µM for 15 min, at 37 °C/5% CO_2_. After pre-treatment, 3T3-L1 cells were treated or not with Lean Conditioned Medium (CM), Obese Conditioned Medium, or differentiation mix. After seven days, the images were registered in an optical microscope (**A**) or lipid were stained with Oil red O which was quantified in a plate cell reader (**B**). D-H. After six days the medium was removed, and cells were incubated with DMEM 1% FBS, for 24 h (to complete seven days). TNFα (**C**), IL-6 (**D**), IL-1β (**E**), leptin (**F**), and adiponectin (**G**) were measured in supernatants using a sandwich enzyme-linked immunosorbent assay kit. Results are representative of three independent experiments. B-H. Data are expressed as means ± SEM. * Represents *p* < 0.05, ** represents *p* < 0.01, **** represents *p* < 0.001.

Having observed increased levels of C/EBPα, we analyzed whether the CM derived from obese AT could induce in 3T3-L1 preadipocytes the morphologic changes required for the differentiation process. In Figure 5A, we show that 3T3-L1 preadipocytes treated with CM derived from obese AT underwent morphologic changes toward a round shape that was similar to the shape of the completely differentiated adipocytes found in the positive control cells (treated with the differentiation cocktail). No changes in morphology were observed in the preadipocytes that were treated with CM derived from lean AT (Figure 5A). To investigate if TLR4 would play a role in the effect of obese AT secretion on the adipocyte differentiation, 3T3-L1 cells were treated with CM derived from obese AT or with the differentiation cocktail, and lipid accumulation was evaluated by oil red O staining. We observed that both treatments increased lipid accumulation within the cells. However, the inhibition of TLR4 signaling by TAK prevented lipid accumulation induced by the CM from obese AT, but no effect was detected in 3T3-L1 cells treated with the differentiation cocktail (Figure 5B). Furthermore, to better characterize the profile of differentiated 3T3-L1, we evaluated the release of proinflammatory and anti-inflammatory adipokines. We observed that the treatment of 3T3-L1 preadipocytes with the CM derived from obese AT, for seven days, stimulated the release of increased levels of TNF-α, IL-6, and IL-1β (Figure 5C–E, respectively), which were eradicated by TAK pre-treatment. Furthermore, we also observed an increase in the release of leptin and adiponectin, both markers of mature adipocytes, which was abolished in the presence of TAK (Figure 5F,G, respectively). Importantly, the treatment with TAK did not inhibit the release of leptin and adiponectin by adipocytes differentiated in the presence of the differentiation cocktail (Figure 5F,G, respectively), indicating that independent pathways may be triggered by the CM and differentiation cocktail.

## 4. Discussion

Inflammation due to dysfunctional AT is a central process involved in the etiopathogenesis of obesity as a hallmark of various metabolic syndrome-associated chronic pathologies [25].

Here, we provide evidence of how the secretion released by obese AT act directly (in a paracrine/autocrine manner) in establishing inflammation on preadipocytes. It should be noted that 15–50% of the cells in AT are preadipocytes [5], which, in obesity, can express increased proinflammatory protein levels [26]. Furthermore, they play a role in the release of inflammatory adipokines involved in the pathogenesis of obesity [27]. Preadipocytes have an inflammatory nature; they became inflamed in response to stimuli such as LPS [28,29] and factors secreted by macrophages [30,31]. Despite evidence that saturated FFAs give rise to inflammation in AT, the contribution of preadipocytes to this effect has yet to be elucidated. Dordevic et al. demonstrated that the FFA exposure for 2 h induced an inflammatory gene expression response, leading to MCP-1 release by preadipocytes [32]. This is an interesting study; however, their study does not mimic the whole pathophysiological process established in obese AT, given the fact that both saturated and unsaturated FFAs are released by obese AT and act in a paracrine manner. Palmitate, oleate, and linoleate are described as the most common FFAs in human fat tissue [5]. Here, we demonstrated that the AT of obese mice releases a wide range of FFAs, such as myristic, palmitic, linoleic, oleic, stearic, and arachidonic acids. Thus, it is crucial to observe the effect of this secretion on the inflammatory profile of preadipocytes. Some studies have already demonstrated the role of some FFAs acting individually on preadipocytes; however, as “adipogenic” cocktails, they do not reflect the conditions in which enlarged fat tissues exist. Guo et al. demonstrated that palmitate increases apoptosis in 3T3-L1 preadipocytes, which is attenuated by the co-treatment with unsaturated fatty acids (oleate and linoleate) [5]. Herein, we aimed to mimic the synergistic pathophysiological role triggered by the total FFAs together with the adipokines released by obese AT on preadipocytes.

Studies have demonstrated that NF-ĸB activation has a role to play in the inflammatory processes in 3T3-L1 preadipocytes. Moreover, NF-ĸB is a key molecule of the TLR4 signaling pathway. Once we had established that FFAs can activate TLR4, we examined the NF-ĸB activation in 3T3-L1 preadipocytes after treatment with the CM derived from obese AT. The results showed that the CM derived from obese AT, which contains several saturated and unsaturated FFAs, enhances the NF-ĸB activation to the same extent as the TLR4 agonist LPS. This effect was blocked when the cells were pretreated with TAK, demonstrating the pivotal role of TLR4 in this process. TLR4 mediates lipid-induced insulin resistance, even though some studies on TLR4-deficient mouse models report controversial results, and TLR4 seems to play an essential role in AT inflammation and insulin sensitivity [33]. Accordingly, it was demonstrated that deletion of the TLR4 gene protects mice from diet-induced insulin resistance, despite an increase in weight gain compared to the control [13]. In contrast, a study conducted in C3H/HeJ mice, which have a spontaneous TLR4 loss-of-function mutation, demonstrated that these mice are protected from diet-induced insulin resistance and weight gain [34]. Similarly, TLR4 mediates ceramide-mediated insulin resistance [35] and inhibition of TLR4 eliminates oxidative stress induced by palmitic acid in endothelial cells [36]. Since these studies used total body knockout or mutant mice, it was not clear whether it was TLR4 on hematopoietic cells or in the AT that promoted the development of insulin resistance. Conversely, a study demonstrated that total body TLR4 knockout or the deletion of TLR4 in non-hematopoietic or hematopoietic cells further enhanced insulin resistance [37]. In our study, the mRNA expression of TLR4 was 4.6-fold higher in preadipocytes treated with the CM derived from obese AT than in the preadipocytes treated with the CM derived from lean AT (Figure S3).

We demonstrated that preadipocytes stimulated with 20% of the CM derived from obese AT presented increased ROS production, which was eliminated by the pre-treatment with TAK, reinforcing the role of TLR4 in the impaired response of preadipocytes in the obese AT milieu. The use of 20% of CM is capable of triggering responses in both primary [16] and immortalized cells [38]. Despite the action of inflammatory cytokines in increasing oxidative stress in obese AT [39], we suggest that the combination of proinflammatory cytokines with FFAs increases ROS observed in obese AT. Asehnoune et al. demonstrated that events in TLR4 signaling are ROS dependent, indicating that ROS can modulate NF-ĸB-dependent transcription via TLR4-mediated responses [40]. Long-chain saturated fatty acids such as palmitic, stearic, and lauric acids are capable of stimulating an inflammatory response through the TLR4 signaling pathway [41,42]. However, according to Lancaster et al., TLR4 is not a receptor for palmitic acid; nevertheless, despite not being a TLR4 agonist, its signaling is TLR4 dependent [43]. Here, we demonstrated that several FFAs are released by obese AT, and considering that both ROS production and NF-ĸB activation were prevented when TLR4 signaling was blocked in 3T3-L1 cells, we reiterate the pivotal role of TLR4 under the inflammatory response in preadipocytes in an obese milieu. Our results further demonstrated that TLR4-dependent signaling is needed to increase the release of IL-1β, IL-6, and TNF-α by preadipocytes stimulated with the CM derived from obese AT.

This study has some limitations. Despite the wide applicability and reliability of 3T3-L1 cells as an in vitro model of adipogenesis, not all signaling pathways are shared with primary preadipocytes. Palmitate itself was not able to induce adipogenic genes in preadipocytes without adipogenic stimuli [5]. FFAs and peroxisome proliferators do not act alone but induce adipogenesis by a shared mechanism, acting synergistically with other inducers to activate adipocyte differentiation [44]. Our results demonstrated that stimulation of preadipocytes with the CM from obese AT induces an increase in C/EBPα levels, as well as leptin and adiponectin release in preadipocytes. We believe that this increase in adipogenic transcription factors is because the CM derived from obese AT is rich in FFAs, which are structurally similar to peroxisome proliferators. Our results strongly suggest that most of the effects observed here were attributable to FFAs, as the denaturation of proteins and the blockade of LPS action with polymyxin induced no changes in lipid accumulation (Figure S2A). However, we cannot rule out that the CM has other participants that may be involved in these processes. Different studies have shown that the obese adipose tissue is capable of releasing adipokines with inflammatory properties [2,4,6,16], which could induce a proinflammatory profile in 3T3-L1 or amplify the FFA effects.

Our data suggest that the secretions released by obese AT act through TLR4, increasing NF-ĸB activity and proinflammatory factors in preadipocytes. Short-term HFD may trigger an acute inflammatory response in AT since it was demonstrated that within three days of HFD feeding, the inflammatory responses are altered in AT [45]. However, macrophages were not crucial in this initial acute inflammatory response as the depletion of macrophages with clodronate did not affect insulin sensitivity in a rodent model of HFD [41]. These results strongly suggest that non-esterified fatty acids are potentially taken up by other cell types in the near vicinity of mature adipocytes, including preadipocytes. Some studies have shown that FFAs induce NF-ĸB in adipocytes [18,19]. In contrast, Cullberg et al. found neither pro- nor anti-inflammatory effects of different FFAs in 3T3-L1 adipocytes [46].

Limited data are available on the differences in inflammatory cytokine expression and activation of NF-κB signaling in preadipocytes, compared with mature adipocytes, following exposure to FFAs, and there are no data showing the paracrine effects of obese AT secretion on preadipocytes. Thus, we propose that inflamed preadipocytes may sustain and exacerbate the low-grade inflammation in obese AT. Recently, Kumar et al. demonstrated that macrophages can be modulated in vitro, by using the CM of senescent or proliferative preadipocytes. While the treatment of proliferative preadipocytes with CM upregulated arginase-1 and mannose receptor genes toward an M2 phenotype, a suppression of these genes was observed when macrophages were cultured in the presence of secretory metabolites of senescent preadipocytes [47]. Preadipocytes from omental fat depots, which possess a high inflammatory profile, induce more monocyte/macrophage infiltration than those from subcutaneous AT [48]. In addition to the inflammatory profile assumed by preadipocytes, we observed an increase in adipogenic transcription factors such as C/EBPα after treatment (for 48 h) with the CM derived from obese AT. These results, together with the increased accumulation of lipids and release of leptin and adiponectin, suggest that these cells differentiated into adipocytes. It is known that adipogenesis is a healthier process than hypertrophy of adipocytes [18,49,50]; however, adipogenesis can lead to inflammation and the release of dangerous FFAs [51]. We observed that even though the CM derived from obese AT induces adipogenesis, these cells are able to secrete increased levels of proinflammatory cytokines TNF-α, IL-6, IL-1β, and leptin in a TLR4-dependent manner, showing that the CM derived from obese AT induces inflammation in preadipocytes, which remains even after differentiation. Our results demonstrated that lipid accumulation in preadipocytes is diminished when cells are pretreated with the antioxidant Trolox (Figure S2B), corroborating the pro-oxidant effects of the obese AT milieu. However, we also observed that seven days after treatment, the differentiated preadipocytes also release increased levels of adiponectin, an anti-inflammatory adipokine [52]. Nevertheless, differentiated adipocytes stimulated with FFAs (oleic, palmitic, palmitoleic, and stearic acids) release increased levels of adiponectin [19,53].

**Figure 6:**
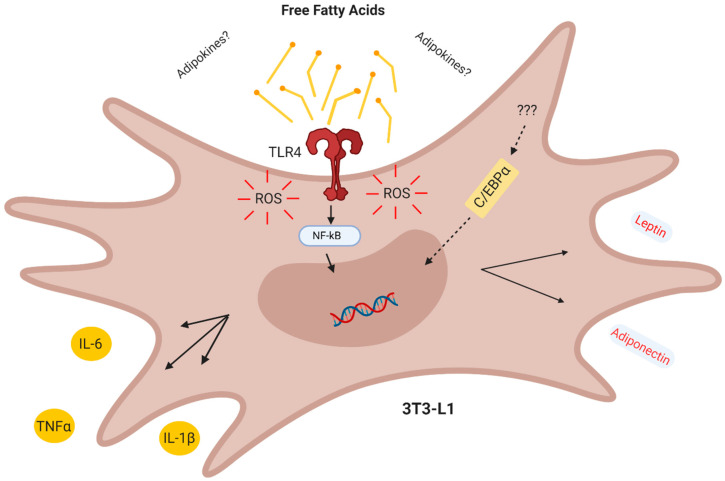
Conclusion. The obese adipose tissue secretes lipokines which act in a combined paracrine/autocrine manner inducing inflammation in preadipocytes via TLR4 and increased ROS, thus creating a paracrine loop which facilitates the differentiation of adipocytes with a proinflammatory profile.

In conclusion, our results demonstrate that in the CM derived from obese AT act in a combined paracrine/autocrine manner inducing inflammation in preadipocytes via increased TLR4 signaling and ROS production, thus creating a paracrine loop, which facilitates the differentiation of preadipocytes to adipocytes with a proinflammatory profile (Figure 6).

## Supplementary Materials

The following are available online at https://www.mdpi.com/2072-6643/12/9/2828/s1, Figure S1: 3T3-L1 cells were treated or not with Lean Conditioned Medium (CM), Obese CM, LPS 1 µg/mL or DMEM 10% FBS at 37 °C/5% CO_2_. Figure S2: 3T3-L1 cells were left untreated or were pre-incubated with A. Polymyxin or B. Trollox 100 µM at 37 °C/5% CO_2_. Figure S3: 3T3-L1 cells were treated or not with Lean Conditioned Medium (CM), Obese CM or LPS 1 µg/mL for 24 h at 37 °C/5% CO_2_. Table S1: Composition of the diets.
