# Supplementary material for: Obese Adipose Tissue Secretion Induces Inflammation in Preadipocytes: Role of Toll-Like Receptor-4
Source: Nutrients. 2020 Sep 16;12(9):2828. doi: 10.3390/nu12092828 (PMC7551792; doi:10.3390/nu12092828)
Supplement: Supplementary file 1 [file nutrients-12-02828-s001.pdf]

nutrients

MDPI

Article

Obese adipose tissue secretion induces inflammation in preadipocytes: role of Toll-like receptor-4

Supplementary information

Table S1. Composition of the diets

Component	Control	HFD
Corn Starch	39	18.5
Dextrose	13.2	6.1
Casein	20	20
Sucrose	10	10
Soybean oil	7	7
Celulose	5	5
Minerals	3.5	3.5
Vitamins	1	1
L-cystine	0.3	0.3
Choline Bitartrate	0.25	0.25
Lard	0	13.3
Carbohydrate	63	34
Protein	20	20
Lipids	7	20.2
Energy density (Kcal/g)	3.9	4.7

*components are expressed as g/100g.

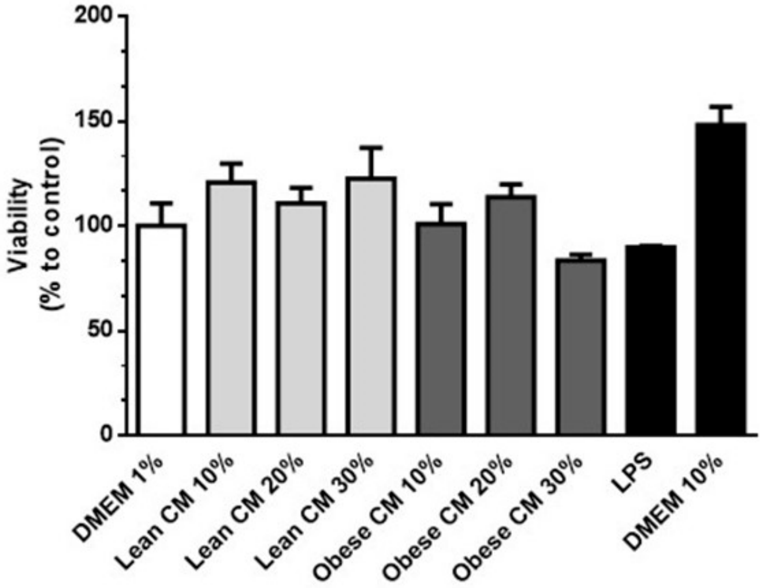

Figure S1. 3T3-L1 cells were treated or not with Lean Conditioned Medium (CM), Obese CM, LPS 1 μg/mL or DMEM 10% FBS at 37º C/5% CO2. After 20 h of treatment, MTT was added and its metabolite was analyzed after 4 h in a microplate reader at wavelength of 570 nm. Results are representative of three independent experiments. Data are expressed as means ± SEM.

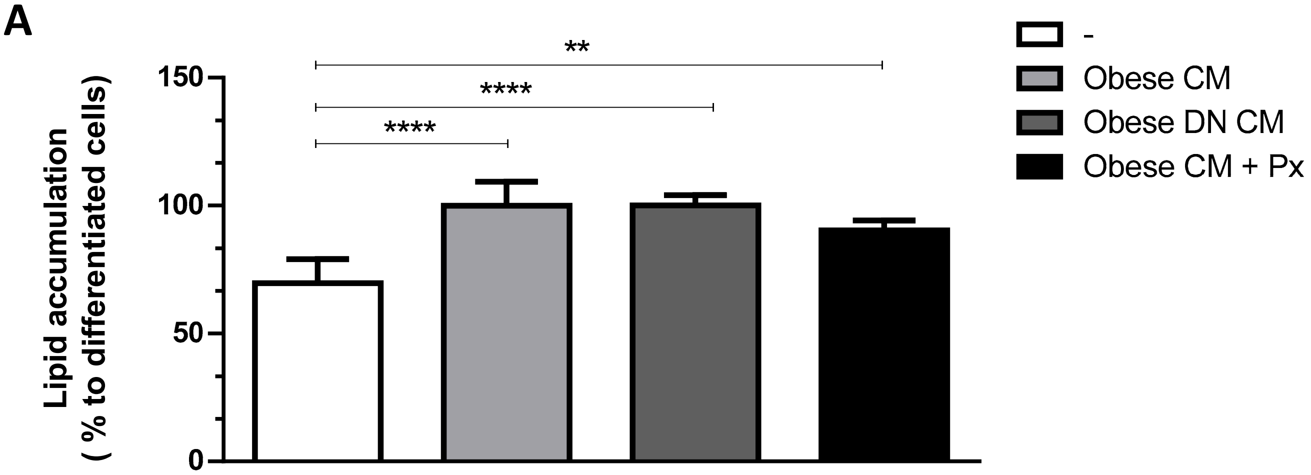

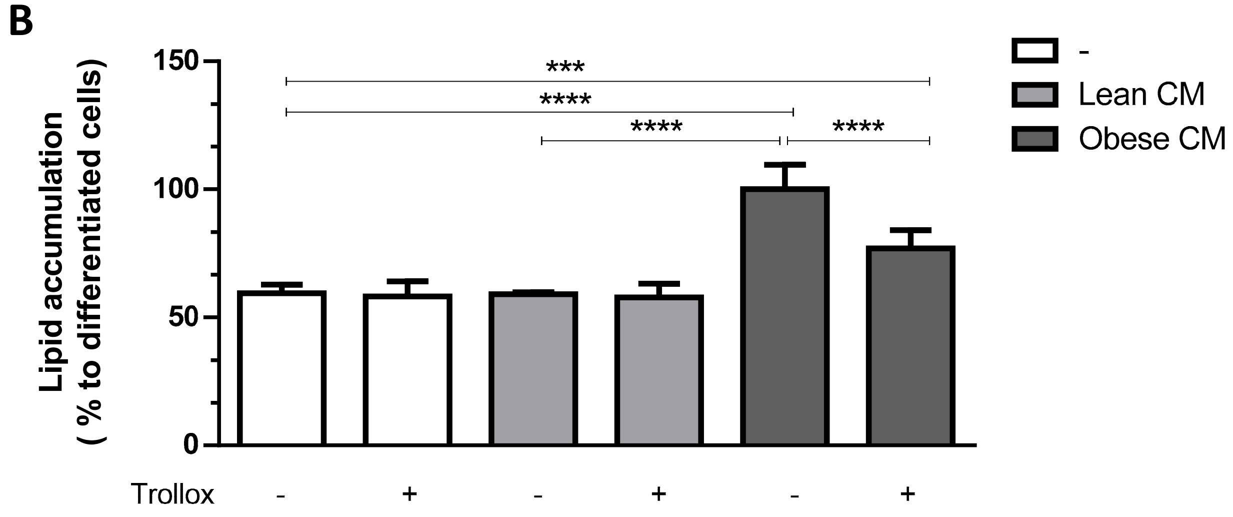

Figure S2. 3T3-L1 cells were left untreated or were pre-incubated with **A.** Polymyxin or **B.** Trollox 100 μM at 37º C/5% CO_2. After pre-treatment 3T3-L1 were treated or not with Lean Conditioned Medium (CM), Obese Conditioned Medium or **A.** Denatured CM. After seven days lipids were stained with Oil red O which was quantified in a plate cell reader. Results are representative of three independent experiments. Data are expressed as means ± SD. ** represents $p < 0.01$, *** represents $p < 0.005$, **** represents $p < 0.001$.

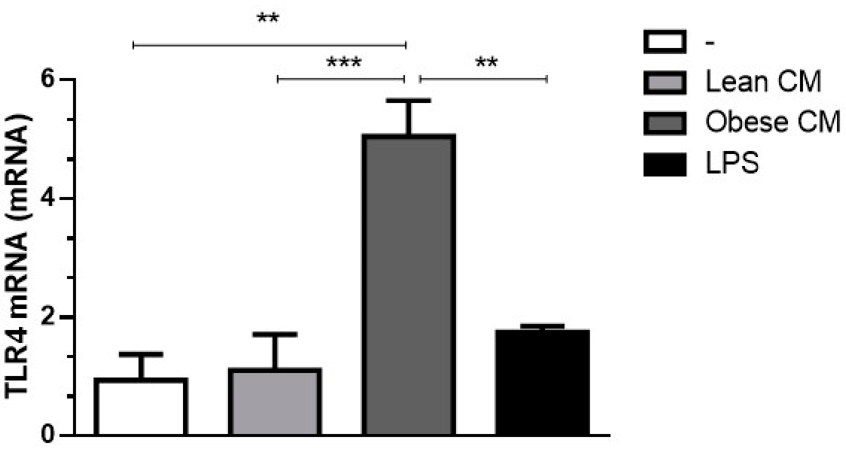

Figure S3. 3T3-L1 cells were treated or not with Lean Conditioned Medium (CM), Obese CM or LPS 1 μg/mL for 24h at 37º C/5% CO_2. TRLR4 mRNA was assessed using qPCR. Amplification of ACTB was performed as endogen control. Data are expressed as means ± SEM. ** represents p< 0.01, *** represents p< 0.005.

© 2020 by the authors. Submitted for possible open access publication under the terms and conditions of the Creative Commons Attribution (CC BY) license (http://creativecommons.org/licenses/by/4.0/).
